# Bioinspired hybrid optimisation and deep belief neural networks for early chronic kidney disease detection: an explainable clinical AI framework

**DOI:** 10.1080/0886022X.2026.2624917

**Published:** 2026-03-15

**Authors:** Zohaib Ahmad, Waeal J. Obidallah, Muhammad Shafiq, Mubarak Albathan, Riyad Almakki, Zeyad Alshaikh, Anas Bilal

**Affiliations:** a Department of Criminology, Lahore Garrison University, Lahore, Pakistan; b College of Computer and Information Sciences, Imam Mohammad Ibn Saud Islamic University (IMSIU), Riyadh, Saudi Arabia; c School of Computer Science, Shandong Xiehe University, Jinan, China; d College of Information Science and Technology, Hainan Normal University, Haikou, China

**Keywords:** Chronic kidney disease, deep learning, bioinspired optimization, feature selection, Explainable AI, clinical decision support

## Abstract

The early detection of chronic kidney disease (CKD) can lead to timely and appropriate clinical intervention. However, most CKD diagnostic systems rely on redundant features and provide unreliable results in small and unbalanced datasets. Therefore, it is challenging to apply them in clinical settings due to a lack of transparency, as well as the extensive amount of time and effort to fine-tune them using manual labor. This article outlines an algorithm to test CKD automatically with a hybrid spiral search strategy-based binary gravitational search algorithm (SSS-BGSA) with elephant herding optimization (EHO) to optimize a Deep Belief Neural Network (DBNN). The code pipeline was designed to involve automated feature selection and model parameter optimization. The objective of using SSS-BGSA in the study was to enhance the exploration-exploitation tradeoff of the original BGSA by a non-linear spiral search pattern. Training of the DBNN using the selected features and optimization of the DBNN parameters were also done using EHO to enhance convergence rate and learning efficiency. The framework was evaluated on the UCI CKD dataset (25 attributes) using nested stratified 5-fold cross-validation. The suggested SSS-BGSA-EHO-DBNN demonstrated a competitive performance (Accuracy = 0.973 ± 0.022, AUC =0.996 ± 0.006), as well as identifying a minimum of 7 clinically important features. Specific gravity, hypertension, packed cell volume, glucosuria, and blood urea were identified as the most important features. The proposed SSS-BGSA-EHO-DBNN framework demonstrates that dual-stage optimization combined with explainable deep learning can yield a fully automated, interpretable, and computationally efficient CKD screening pipeline.

## Graphical Abstract

**Figure:**
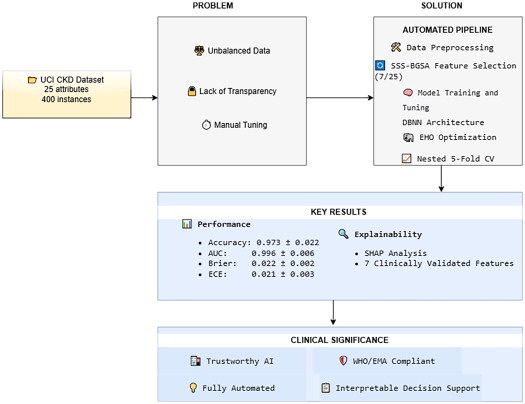

## 1. Introduction

CKD has been reported to affect more than 10% of adults worldwide and remains a public health problem [1,2]. CKD exerts a major strain on medical systems by raising healthcare costs, leading to serious complications, and causing increased mortality. The insidious progression of CKD, with often no notable symptomatology in early stages, is one of the major challenges in the diagnosis of CKD. Screening for renal dysfunction has traditionally relied on serum creatinine and estimated glomerular filtration rate (eGFR), which are often non-sensitive to early renal impairment [3]. This highlights an urgent need for the development of innovative diagnostic methods with the potential of incorporating intelligent data-driven methods to identify CKD earlier.

Significant advancements have occurred in artificial intelligence (AI) recently, suggesting enormous potential to improve current CKD diagnosis systems. The applications based on AI that utilize machine learning (ML) and deep learning (DL) deliver the ability to identify non-linear relationships within medical data that cannot be identified by standard methods of diagnosis [4,5]. These approaches have shown enhanced accuracy for early CKD detection and improved patient outcomes. For instance, previous studies using decision trees, support vector machines (SVMs), and deep neural networks (DNNs) have achieved promising results on CKD datasets [5–7] achieved a classification accuracy of 98.5% using a deep belief neural network (DBNN), while the authors in [8] achieved over 94% with a stacked autoencoder. Similarly, ensemble approaches such as XGBoost combined with recursive feature elimination have enhanced early detection accuracy [6].

Recent advancements in the integration of metaheuristic optimization algorithms with DL have also improved the ability of medical diagnostic systems. In keeping with this approach, the authors [9] validated that pairing a firefly-based feature selector with a neural network can achieve a better CKD classification. On the other hand, the authors [10] presented a hybrid Binary Grey Wolf Optimization (BGWO) with an Extreme Learning Machine (ELM) for feature selection and classification. These studies highlight the worth of combining bioinspired optimization with deep models to boost feature discrimination, reduce redundancy, and enhance generalization competency on small medical datasets.

Nevertheless, despite this progress, some barriers continue in applying ML/DL practices for CKD diagnosis, mostly in fulfilling standards for trustworthy and reproducible clinical AI as defined by international health and regulatory agencies [11,12] and leading nephrology societies [13].

- Low interpretability and overfitting risks due to the high dimensionality and limited size of CKD datasets. Studies [9,14–16] show that hybrid metaheuristic feature selection can effectively identify informative variables and reduce redundancy, thereby improving generalization.
- Class imbalance remains a critical issue, as CKD-positive samples are often underrepresented. Oversampling approaches such as SMOTE [17] and its adaptive variant ADASYN [18] are widely employed to improve model sensitivity and mitigate bias toward negative cases.
- Missing data is another major concern in clinical datasets. Classical imputation methods [19] may not preserve complex relationships. Advanced hybrid techniques such as iterative imputation [20], generative models [21], and multivariate imputation by chained equations (MICE) [22] are increasingly applied to enhance data completeness and model robustness.

### 1.1. Proposed contribution

To overcome the above constraints, this research proposes a bioinspired hybrid system that involves metaheuristic feature selection and optimized DL to provide strong CKD diagnosis. Nonetheless, some of the known studies emphasize primarily on maximizing accuracy; our work highlights the scale of clinically translatable AI pipeline to balance high-performance and the real-life demands of actual implementation: automation, interpretability, and efficacy.

Implicitly, a novel Spiral SSS-BGSA of automated feature selection is proposed, and combined with an EHO-tuned DBNN. The essential functions of this combined SSS-BGSA-EHO-DBNN system are:

- Unlike conventional approaches that require manual feature engineering and hyperparameter tuning, our framework automates both processes through bioinspired optimization, enhancing reproducibility and reducing the dependency on expert intervention.
- The framework incorporates SHAP-based Explainable AI (XAI) to provide transparent feature importance rankings. The selected biomarkers (e.g., specific gravity, hypertension, packed cell volume) are validated against established clinical guidelines [23]), ensuring the model’s decisions are clinically coherent and trustworthy.
- By identifying a minimal set of highly discriminative features, the model reduces data acquisition costs and computational overhead, facilitating easier integration into clinical workflows.
- Furthermore, the framework incorporates probability calibration assessment to ensure that predicted risks align with observed outcomes, a critical requirement for clinical screening tools.

By accomplishing competitive performance with state-of-the-art approaches through this automated and interpretable pipeline, our work delivers a major step concerning bridging the gap between high-accuracy AI research and deployable clinical decision-support systems. This approach supports the framework with both clinical practice guidelines [23] and international standards for trustworthy AI in healthcare [11,12] and recent consensus statements on AI application in nephrology [13].

Paper organization: The rest of this paper is structured as follows: Section 2: A structured review of related work on CKD diagnosis, data quality challenges, ML and DL models, imbalance-handling strategies, and bioinspired feature-selection methods. The proposed framework of SSS-BGSA–EHO–DBNN is described in Section 3, which includes the dataset preprocessing pipeline, the metaheuristic feature selection mechanism, the DBNN optimization, and the integration of SHAP-based explainability. The experiments described in Section 4 include a description of the dataset, model evaluation, and comparison against state-of-the-art methods, followed by a specific discussion of clinical interpretability (*via* SHAP analysis) and dedicated regulatory compliance assessment (aligning with WHO [11] and EMA [12] guidelines). Section 5 discusses concluding remarks, contributions, and potential applications for the use of the framework in AI-assisted CKD systems. The list of the abbreviations used in this study are given in Table 1.

**Table 1.** List of the abbreviations.

Abbreviation	Definition
AI	Artificial intelligence
AUC	Area under the ROC curve
BGSA	Binary gravitational search algorithm
BGWO	Binary grey wolf optimization
CKD	Chronic kidney disease
DBNN	Deep belief neural network
EHO	Elephant herding optimization
EHR	Electronic health record
EMA	European medicines agency
GSA	Gravitational search algorithm
MICE	Multivariate imputation by chained equations
RBM	Restricted Boltzmann machine
SHAP	SHapley additive exPlanations
SMOTE	Synthetic minority over-sampling technique
SSS-BGSA	Spiral search strategy-based binary gravitational search algorithm
WHO	World health organization
XAI	Explainable artificial intelligence
KDIGO	Kidney disease: improving global outcomes
ADASYN	Adaptive synthetic sampling
ECE	Expected calibration error

## 2. Literature survey

Missing values and class imbalances are two data quality issues that impair both model dependability and diagnostic accuracy in CKD datasets, mainly those from the well-known UCI collection. Because of the wide disparity between CKD cases and non-CKD cases in clinical cases, this can lead to classifiers being biased toward the majority class. Previous methods of oversampling, including SMOTE [17], which is still a common method, and newer hybrid methods like SMOTE-ENN, where oversampling is correlated with Edited Nearest Neighbors (ENN) noise reduction [24], have shown improvement in the resilience of a classifier. In addition to the previously mentioned methods, other techniques include cluster-based undersampling and Tomek link refinement, which may be used to reduce overlap between classes and improve fairness for high-stakes classification [25]. While some of these methods show promise, they require an integrated approach when using them in large-scale diagnostic processes in order to minimize data leakage or over-smoothing.

The second inquiry into clinical CKD databases is missing information. Traditional approaches for filling the missing data with mean, median, and mode substitution methods fail to maintain non-linear relationships among clinical variables. The latest contribution has therefore been on the formulation of new model-based adaptation methods to cater to missing data. Attention-based matrix completion has proved to be better than other models in the reconstruction of missing data by identifying of relationship of cross features [26]. Estimation of laboratory values related to CKD has also been performed by utilizing temporal estimation with bidirectional GRU networks [27]. The application of these procedures provides an extra layer of reliability to clinical CKD database preprocessing.

Although earlier versions of ML algorithms, such as traditional decision tree classifiers, support vector machines (SVM), logistic regression, K-nearest neighbors, were researched for the early detection of CKD due to their ease of interpretation, they produced very poor results on large, high-dimensional datasets and datasets containing excessive amounts of noise. Due to these factors, many researchers utilized the UCI CKD dataset for experiments and after completing careful preprocessing on the data, reported accuracy rates from 88 to 92% [28]. In addition to its ability to handle higher levels of noise, the authors in [29] also pointed out that Random Forests are appropriate models for modeling nonlinear relationships between variables.

Gradient-boosting-based models (e.g. AdaBoost, XGBoost, CatBoost, and LightGBM) have been shown through recent research to outperform traditional classifier types for predicting CKD by providing significant increases in performance. Gradient boosting-based model results have shown consistent accuracy rates of above 91%, and accuracy has reached as high as 98.47% using the UCI CKD dataset [30,31]. Although gradient boosting is a powerful method of prediction, there are many other challenges that remain to be addressed regarding the use of gradient boosting models. Specifically, all gradient boosting models require extensive amounts of manual data preprocessing, heuristic feature selection, and substantial amounts of time to perform hyperparameter tuning. These additional steps required before being able to utilize these models make it very difficult to automate, replicate and apply to the real-world clinical environment.

Therefore, DL has become an increasingly popular method to predict CKD due to the success of several approaches utilizing convolutional neural networks (CNNs), recurrent neural networks (RNNs), and specifically combined CNN-LSTM ensemble models in detecting early-stage CKD [32]. Most recently [32], developed a DL-based model called “Deep-Kidney,” which has shown promising results as it learns directly from clinical features and achieved strong predictive performance for CKD detection. DBNNs constructed from stacked Restricted Boltzmann Machines (RBMs) have also shown strong capabilities in learning hierarchical feature representation of CKD data, and the authors in [5] reported a high accuracy of 98.5% when a DBNN was used for predicting CKD, supporting the use of this architecture for the prediction of CKD in small, structured medical datasets. The DL models, however, will suffer performance degradation if the hyperparameters of the model are not optimized appropriately. Therefore, the motivation exists for incorporating automated, bio-inspired tuning methods into the process of optimizing the hyper-parameters of deep architectures.

Explainability has emerged as a vital component for deploying AI systems in the healthcare space. The crucial responsibility of clinicians is to guarantee transparency concerning the relevance of features and the decision-making procedure, expressly for high-risk health situations such as CKD. Global and local explanations through methods such as SHAP have become a prevalent approach to offer clinicians an explanation for their models’ predictions. The study by the authors [33] supported the efficacy of SHAP in presenting a global explanation of CKD predictive models. This study also increases clinician’s reliance on such models. The conclusions of this study support the importance of clinical diagnostic frameworks, which provide a direct link between the accurate diagnosis and the clear, transparent, and clinically relevant explanations of the reasoning supporting the diagnosis.

Selecting the most important features for modeling improves the model’s interpretability, improves the model’s ability to generalize, and reduces the computational requirements needed for training the model. Various meta-heuristics, including Binary Whale Optimization Algorithm (BWOA), Grey Wolf Optimizer (GWO), Cuckoo Search Optimization (CSO), and Ant Lion Optimizer (ALO), have been engaged efficaciously to find smaller but highly discriminatory feature sets from large feature spaces. For instance, using BWOA to select from a set of clinical biomarkers for CKD resulted in an improvement in classification accuracy by more than six percent when compared to SVMs [34]. These studies are also consistent with the results of [35], which demonstrated that ALO achieved a reduction in dimensionality greater than 30% while maintaining model performance. The results from CSO, GWO, and other bioinformatics applications for example kidney biomarkers [10,36,37], suggest that this type of optimization can be useful in examining a number of features within a high-dimensionality clinical data set with a relatively limited number of samples, and also to find an optimal balance between exploration and exploitation of the search space by using different types of metaheuristics.

Automated medical diagnosis through hybrid approaches of DL and metaheuristics has freshly gained increasing attractiveness. A recent research by [38] suggested a framework to diagnose CKD that engaged a combination of the simulated annealing method to select features with CatBoost to classify patients. An additional hybrid methodology was established by [39] using adaptive particle swarm optimization (APSO) to optimize echo state networks (ESNs) that attained a classification accuracy of 99.6% when trained and tested against the MIMIC III dataset. At the same time, a 2023 Big Data & Cognitive Computing study [40] utilized iterative imputation to fill missing values prior to applying Boruta feature selection and utilizing a k-nearest neighbors model to identify CKD. The hybrid model illustrates that combining automated methods for feature selection and hyperparameter tuning in conjunction with robust pre-processing can provide superior accuracy in identifying CKD from high levels of noisy and/or imbalanced medical data.

Despite these advances, several limitations persist in the current literature:

- Most studies treat preprocessing, feature selection, and classifier construction as isolated stages rather than integrating them into a unified optimization framework.
- Many metaheuristic algorithms can suffer from premature convergence or ineffective feature exploration, mainly when working with small datasets, such as CKD.
- Few models consider explainability approaches or assess compliance with international guidance for trustworthy medical AI [11,12].

In order to address the identified limitation, this study presents a unified bio-inspired diagnostic framework which combines an SSS-BGSA for auto-feature extraction with an EHO-tuned DBNN as well as SHAP explainability, to ensure that the results are both transparent and clinically meaningful. The proposed integrated diagnostic framework aims to eliminate feature redundancy, enhance generalization capabilities on small imbalanced data sets, and ultimately provide a reproducible and reliable clinical AI pipeline.

## 3. Materials and methods

### 3.1. Data set

The proposed model uses the UCI Dataset [41]. It consists of 25 attributes, 14 nominal, 11 numeric and 1 class attribute, which represent the data of 400 patients, out of which 250 have CKD and 150 are not. Table 2 shows the description of the used dataset.

**Table 2.** Data set description of CKD.

ID	Attribute	Abbreviation	Attribute Category	Scale
f1	Age	age	Numerical	Years
f2	Blood pressure	bp	Numerical	mm/Hg
f3	Specific gravity	sg	Nominal	{1.005, 1.010, 1.015, 1.020, 1.025}
f4	Albumin	al	Nominal	Semi-quantitative (0–5)
f5	Sugar (urine glucose)	su	Nominal	Semi-quantitative (0–5)
f6	Red blood cells	rbc	Nominal	{normal, abnormal}
f7	White blood cells (pus cells)	pc	Nominal	{normal, abnormal}
f8	White blood cell clumps (pus cell clumps)	pcc	Nominal	{present, not present}
f9	Bacteria	ba	Nominal	{present, not present}
f10	Blood glucose random	bgr	Random Numerical	mg/dL
f11	Blood urea	bus	Numerical	mg/dL
f12	Serum creatinine	sc	Numerical	mg/dL
f13	Sodium	Sod	Numerical	mEq/L
f14	Potassium	pot	Numerical	mEq/L
f15	Hemoglobin	hemo	Numerical	g/dL
f16	Packed cell volume	pcv	Numerical	%
f17	White blood cell count	wbcc	Numerical	cells/cmm
f18	Red blood cell count	rbcc	Numerical	millions/cmm
f19	Hypertension	htn	Nominal	{yes, no}
f20	Diabetes mellitus	dm	Nominal	{yes, no}
f21	Coronary rrtery disease	cad	Nominal	{yes, no}
f22	Appetite	appet	Nominal	{good, poor}
f23	Pedal Edema	pe	Nominal	{yes, no}
f24	Anemia	ane	Nominal	{yes, no}
f25	Class label (CKD status)		Nominal	{ckd, notckd}

### 3.2. Methodology

This study proposes a robust three-stage framework for CKD diagnosis: data preprocessing, feature selection, and classification.

**Figure 1.**
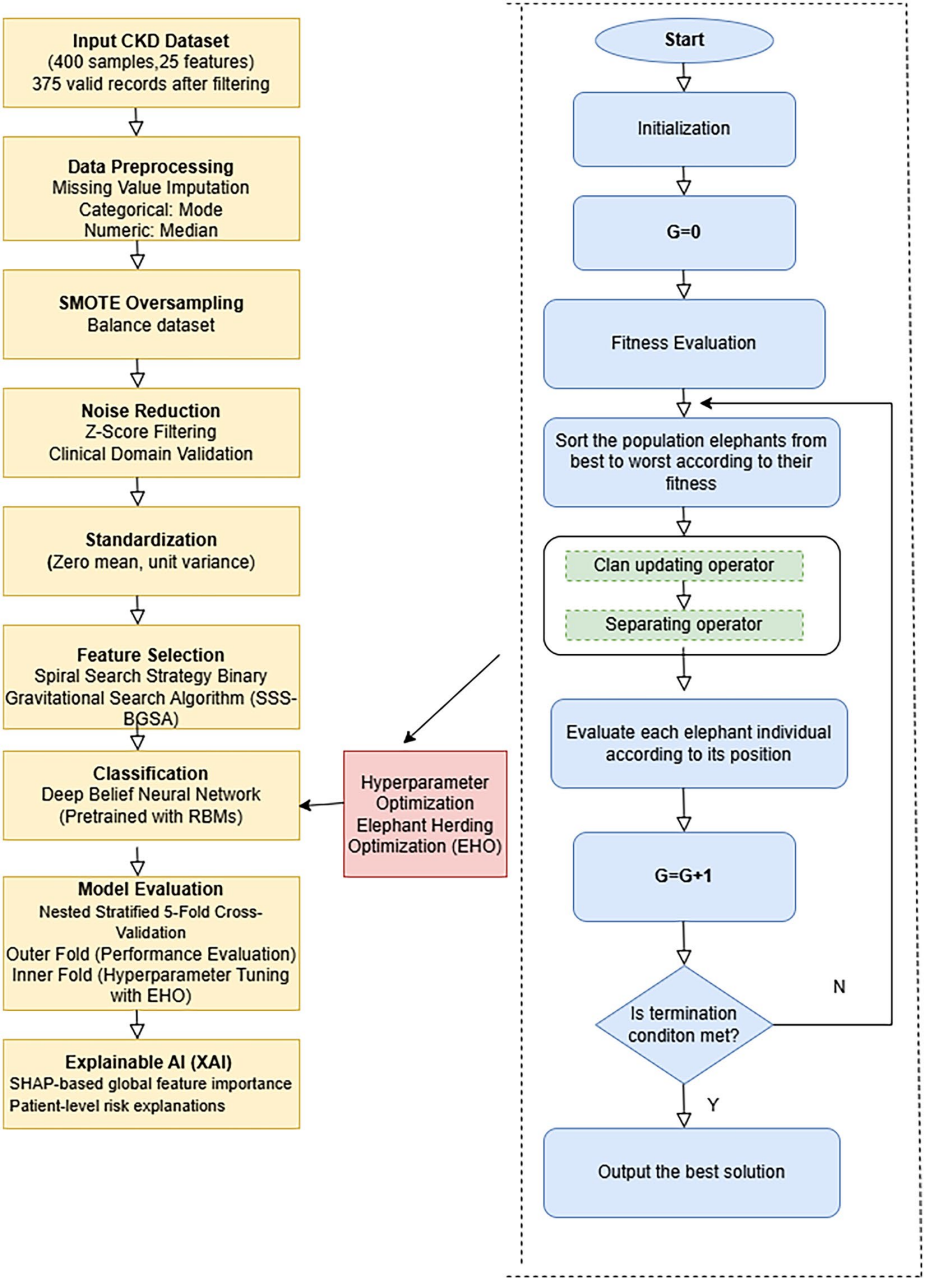
Overview of the proposed framework.

Figure 1 explains the schematic diagram of the system.

### 3.3. Data preprocessing

Data preprocessing is the primary and most vital step of the pipeline, to ensure the data quality, consistency, and suitability to train a model and evaluate it appropriately. Data preprocessing entails; missing value management, class balancing, noise reduction and standardization.

#### 3.3.1. Handling missing values

Medical datasets often include missing values which may influence model’s performance. Following best practices defined by [19,42], missing values are imputed by domain-specific approaches. The imputation parameters analyzed from the training data and then applied to the validation/test sets to avoid data leakage:

- Categorical features (e.g., red blood cells, pus cells): Missing values are imputed using mode imputation, which replaces missing entries with the most frequent category in the feature:
(1) $$\text{Mode}\left(f\right)=\begin{array}{c} \text{arg}\;\text{max}\\ c\epsilon C \end{array}\sum_{i=1}^{n}(x_{i}=c)$$

where, C signifies a set of unique categories in feature f and $x_{i}$ signifies the ith observation.

- Numerical Features (e.g., serum creatinine, blood pressure): Missing values have been imputed using the median to reduce the effect of outliers:
(2) $$\text{Median}\;\left(f\right)=\left\{\begin{array}{l} x_{\left(\frac{n}{2}\right),}\\ \underline{x_{\left(\frac{n}{2}\right)}+x_{\left(\frac{n}{2}+1\right)}}\\ 2 \end{array}\right.,\;\begin{array}{c} \text{if}\;n\;\text{is}\;\text{odd}\\ \text{if}\;n\;\text{is}\;\text{even} \end{array}$$

where x signifies the sorted vector of observed values.

- Instance Removal: Instances with more than 50% missing values are removed to prevent data bias:
(3) $$\sum_{j=1}^{m}\left(x_{\text{ij}}=\text{NaN}\right)\geq 0.5m.$$

Applying this criterion excluded 25 of the original 400 records, yielding a final dataset of 375 instances (230 CKD, 145 non-CKD).

#### 3.3.2. Class balancing

The SMOTE algorithm was applied to the CKD dataset to address class imbalance, as described by the authors [17]. The synthetic samples generated by SMOTE enable interpolation between existing instances of the minority class, reducing bias toward the majority class. Minority-class instances determine their k=5 nearest neighbors through Euclidean distance calculation, as follows:

(4) $$d\left(x_{i},x_{j}\right)=\sqrt{\sum_{l=1}^{m}{\left(x_{\text{il}}-x_{\text{jl}}\right)}^{2}}$$

A new synthetic instance is then generated using

(5) $$x_{\text{new}}=x_{i}+⋋.\left(x_{j}-x_{i}\right),\;⋋∼\mu \left(0,1\right)$$

The choice of SMOTE over ADASYN came from its ability to produce uniform samples that enhance learning stability and diagnostic interpretation in medical settings. The execution of SMOTE relied on imbalanced-learn (v0.11.0) with k−neighbors=5.

To strictly avoid data leakage, SMOTE was applied only on the training portion within each stratified fold; the corresponding validation/test subsets were kept at their original class distribution. Within each training fold, oversampling increased the minority (non-CKD) class so that both classes were approximately balanced; these synthetic samples were used exclusively for model training, while performance was evaluated only on untouched real patient data in the validation and test sets.

#### 3.3.3. Noise reduction

Noise reduction techniques were applied in the data quality improvement on both the numeric features and categorical values.

The Z-score filtering scheme detected outliers, which were removed since numeric values had Z-scores above ±3. The Z-score is calculated by the following formula for a given value:

(6) $$z=\frac{x-\mu }{\sigma }$$

where μ and σ designate the mean deviation and standard deviation of feature f.

For categorical variables, domain validation ensures clinical plausibility. For example, Hypertension is constrained to {“yes”, “no”} based on the WHO diagnostic criteria [11]. Invalid entries (e.g. “?”, “unknown”, misspellings) are corrected using contextual information or removed. Similar validation is applied to red blood cells and pus cells, which are restricted to {“normal”, “abnormal”} in line with nephrology standards [28,43].

Such a combination of clinical and statistical-based cleaning ensured a high-quality data that can be used to build effective predictive models.

#### 3.3.4. Feature scaling and standardization

The model training depends equally on numerical features through the standardization of features. All numerical features transform to establish a zero mean, combined with unit variance:

The model training relies equally on numerical features by the standardization of features. The entire numerical features transform to establish a zero mean, combined with unit variance, as follows:

(7) $$\overset{\prime }{x}=\frac{x-\mu }{\sigma }$$

Parameters (μ) and (σ) are calculated from the training sets only and then applied to the validation/test sets, preventing data leakage. Standardization mitigates the influence of attributes with large numeric ranges (e.g. Age, Hemoglobin). The process is executed using the StandardScaler from scikit-learn [44].

#### 3.3.5. Dataset partitioning

The cleaned CKD dataset was evaluated using a nested stratified cross-validation framework to ensure unbiased performance estimation and robust model selection. In this setup, the outer stratified 5-fold cross-validation loop was used exclusively to assess generalization performance, while the inner cross-validation loop was employed for data preprocessing, feature selection, and hyperparameter optimization.

To avoid optimistic bias and information leakage, all preprocessing steps—including missing-value imputation, SMOTE oversampling, and feature standardization—were performed only within the training folds of the inner loop, and subsequently applied to the corresponding validation subsets. This strategy preserves class proportions across folds and provides a balanced, clinically validated, and standardized data representation, supporting robust and explainable CKD prediction.

### 3.4. Navigating the feature selection phase

Reducing number of input features is a great advantage of feature selection in developing a medical diagnostics pipeline identifying early signs of CKD, the reduction of the irrelevant input features leads to better model interpretability and also improves the predictive accuracy of the models. A bio-inspired hybrid meta-heuristics method, named SSS-BGSA, has been proposed here by integrating the global exploration abilities of the GSA with a logarithmic spiral-based local search technique, which provides an effective method to select the most appropriate subset of relevant features from the original set of input features. This integration enhances the balance between exploration and exploitation, hence mitigating premature convergence in high-dimensional search spaces.

#### 3.4.1. Gravitational search algorithm (GSA)

The optimization method GSA originated from Newtonian gravity and kinematics as defined by [45]. GSA uses modeling of agents as a mass whose attraction to each other depends on their fitness values, enabling population-based optimizers to find promising solution areas.

In feature selection, the fitness of an agent i at iteration t, signified as $f_{i}(t)$, is determined by balancing classification error and feature subset size:

(8) $$f_{i}\left(t\right)=\alpha \;.\;\text{Error}\;\text{Rat}e_{i}\left(t\right)+\beta \;.\;\frac{\left|S_{i}\right|}{\left|D\right|}$$

where, ${\text{Error Rate}}_{i}(t)$ designates the classification error for subset $S_{i}$, $\left|S_{i}\right|$ signifies the number of features nominated by agent i, α,β represent the weighting coefficients (commonly α=0.9, β=0.1), and |D| designates the total number of features.

Gravitational Force and Position Update: The gravitational force $F_{\text{ij}}^{d}(t)$ exerted by agent j on agent i in dimension d is given by:

(9) $$F_{\text{ij}}^{d}=G\left(t\right)\;.\;\frac{M_{i}\left(t\right)\;.\;M_{j}\left(t\right)}{R_{\text{ij}}\left(t\right)+\epsilon }\;.\;(X_{j}^{d}\left(t\right)-X_{i}^{d}\left(t\right)$$

where $M_{i},M_{j}$ represent the masses of agents, $R_{\text{ij}}$ represents the Euclidean distance between agents, and ϵ signifies a small constant to avoid division by zero

Agent acceleration $a_{i}^{d}(t)$ and velocity $v_{i}^{d}(t+1)$ and position $x_{i}^{d}(t+1)$ updates are defined as follows:

(10) $$a_{i}^{d}(t)=\frac{F_{i}^{d}(t)}{M_{i}(t)}$$

(11) $$v_{i}^{d}(t+1)={\text{rand}}_{i}.v_{i}^{d}(t)+a_{i}^{d}(t)$$

(12) $$x_{i}^{d}(t+1)=x_{i}^{d}(t)+v_{i}^{d}(t+1)$$

Although GSA demonstrates strong exploitation capabilities, it often suffers from premature convergence and reduced diversity in high-dimensional feature spaces. To overcome these limitations, the SSS is integrated into the optimization process to enhance exploration potential.

**Figure 2.**
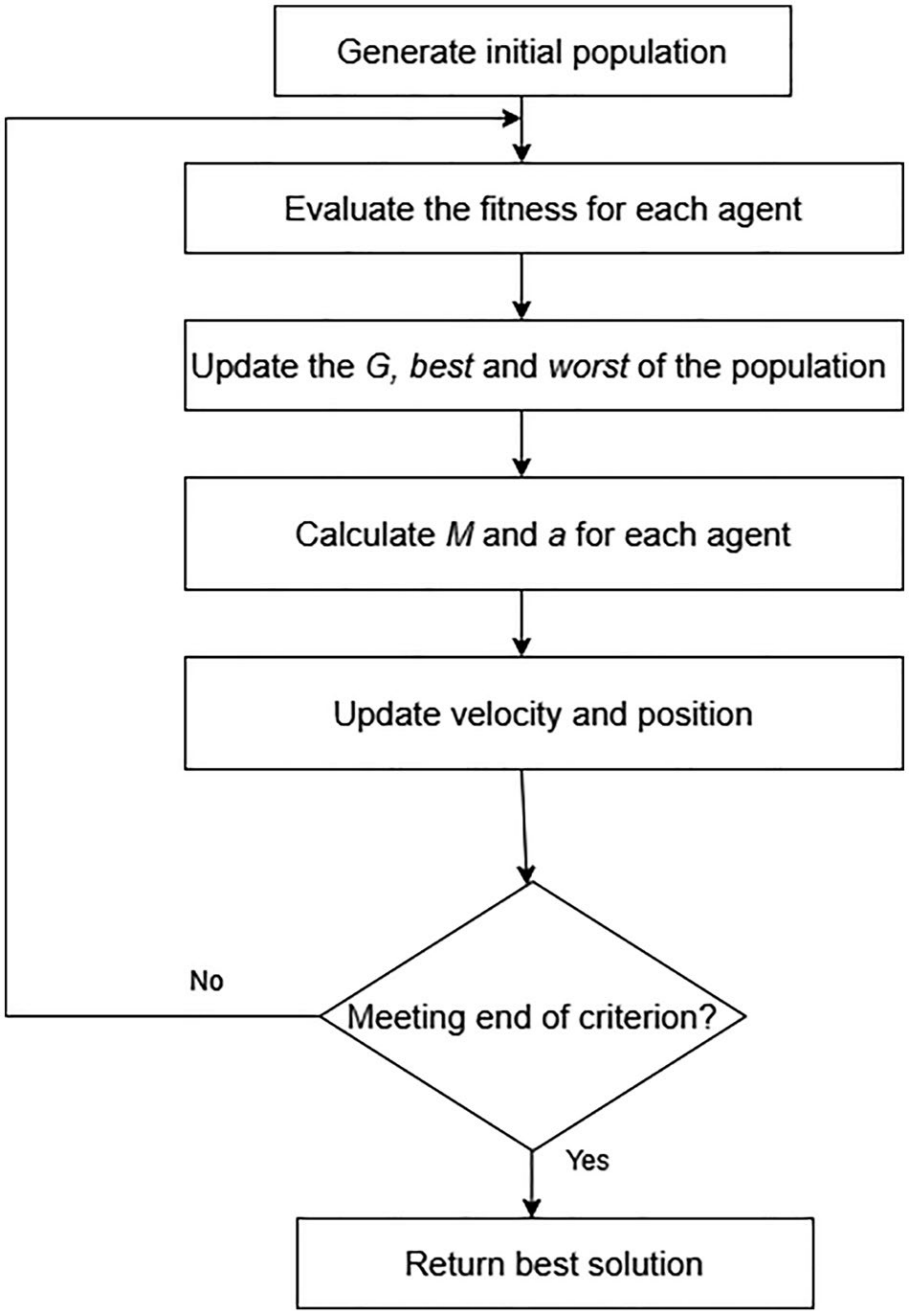
Flow diagram of the Standard GSA.

The flow diagram of the standard GSA is shown in Figure 2.

#### 3.4.2. Spiral search strategy–based binary gravitational search algorithm (SSS-BGSA)

To enhance search diversity and escape local minima, the SSS-BGSA introduces a logarithmic spiral-based motion inspired by moth navigation and spiral galaxy trajectories, both known for adaptive exploration patterns [15,46].

Spiral Search Strategy: The spiral trajectory updates the position of an agent i in dimension d according to the following equations:

(13) $$x_{i}^{d}\left(t+1\right)=x_{i}^{d}\left(t\right)+r\;.\;e^{b\;.\;\theta }\;.\text{ cos}\left(\theta \right)$$

(14) $$y_{i}^{d}\left(t+1\right)=y_{i}^{d}\left(t\right)+r\;.\;e^{b\;.\;\theta }\;.\text{ sin}\left(\theta \right)$$

where: r designates the distance from the spiral center, b represents the spiral expansion constant, and θϵ[0,2π] represents the angular position.

The spiral motion allows particles to route around local optima (thus refining the local search in promising areas).

As the feature selection issue is binary in nature, continuous position updates are transferred to a binary decision using a sigmoid transfer function as below:

(15) $$S(v_{i}^{d}(t))=\frac{1}{1+e^{-v_{i}^{d}(t)}}$$

An agent’s position in dimension d is then updated as:

(16) $$x_{i}^{d}\left(t+1\right)=\left\{\begin{array}{l} 1,\;\text{if}\;S\left(v_{i}^{d}\left(t\right)\right)>\text{ran}d_{i}\\ 0,\;\text{otherwise} \end{array}\right.$$

In contrast to prior spiral-based mechanisms, which were coupled with particle swarm or moth-flame optimizers [15,46], the suggested SSS-BGSA incorporates a logarithmic spiral update in conjunction with the BGSA. The proposed design, a spiral motion operating on agents under GSA’s mass-fitness dynamics, is combined with a binary transfer function to determine whether or not to include features. The combination of these two methods allows agents to orbit promising areas while maintaining GSA’s global search characteristics, which aids in balancing the exploration–exploitation tradeoff and reducing the occurrence of premature convergence in high-dimensional binary feature spaces.

The SSS-BGSA algorithm excellently balances:

- Global convergence: via gravitational attraction in GSA,
- Local exploration: through the spiral search mechanism for escaping stagnation, and
- Binary decision-making: by incorporating a transfer function suited for feature selection.

This hybrid formulation enables the discovery of compact yet diagnostically significant feature subsets, improving classification accuracy, reducing redundancy, and enhancing interpretability for CKD diagnostics. The SSS-BGSA–based feature selection technique is summarized in Table 3.

**Table 3.** SSS-BGSA -Based feature selection technique.

Step	Action
Input	N : Number of agents (population size), D : Total features in CKD dataset, $T_{\text{max}}$ : Maximum iterations, $G_{0}$ : Initial gravitational constant, $w_{\text{max}}$ / $w_{\text{min}}$ : Inertia weight bounds, SP : Spiral search probability
Output	$X_{\text{best}}$ : Optimal binary feature vector (1 = selected, 0 = excluded)
1	Initialize: Generate N agents $\{X_{1},\ldots ,X_{N}\},{\;X}_{i}\epsilon {\left\{0,1\right\}}^{D}$ Set $G\leftarrow G_{0}$ , $w\leftarrow w_{\text{max}}$ , $X_{\text{best}}\leftarrow \phi$ , $f_{\text{best}}\leftarrow +\infty$
2	For t=1 to $T_{\text{max}}$ do
2a	Evaluate cost: For each agent *i*: Train DBNN using features with $X_{i}[d]=1$ Compute ${\;\text{ErrorRate}}_{i}$ Calculate $f_{i}=0.9\;.\;\text{ErrorRat}e_{i}+0.1\;.\;\frac{{\left|S_{i}\right|}_{0}}{D}$ (smaller $f_{i}$ indicates a better subset)
2b	Update Global Best: If $f_{i}<f_{\text{best}}$ , then set $f_{\text{best}}\leftarrow f_{i}$ and $X_{\text{best}}\leftarrow X_{i}$
2c	Update Parameters: Gt←G0 . exp−15.tTmax. w(t)=wmax−(wmax−wmin).tTmax
2d	Compute Masses Let $f_{\text{max}}={\text{max}}_{i}f_{i}$ , $f_{\text{min}}={\text{min}}_{i}f_{i}$ For each agent *i*, $m_{i}=\frac{f_{\text{max}}-f_{i}}{f_{\text{max}}-f_{\text{min}}+\epsilon }$ , $M_{i}=\frac{m_{i}}{\sum_{j=1}^{N}m_{j}}\;$
2e	Calculate Forces & Acceleration: For each search agent *i*, calculate : $F_{\text{ij}}^{d}=G\left(t\right)\;.\;\frac{M_{i}\;.\;M_{j}}{R_{\text{ij}}+\epsilon }\;.\;(X_{j}^{d}-X_{i}^{d})$ $a_{i}^{d}=\frac{\sum_{j\epsilon K_{\text{best}}}F_{\text{ij}}^{d}}{M_{i}}$
2f	Update Velocity & Position (Spiral Search) : $v_{i}^{d}\leftarrow w\left(t\right)\;.\;v_{i}^{d}+a_{i}^{d}$ With probability SP: $X_{i}^{d}\leftarrow X_{i}^{d}+\left(X_{\text{best}}^{d}-X_{i}^{d}\right)\;.\;e^{b\theta }\;.\text{ cos}\left(2\pi \theta \right),\;\theta ∼U(-1,1)$ Else: $X_{i}^{d}\leftarrow X_{i}^{d}+v_{i}^{d}$ Binarize using sigmoid: $p_{i}^{d}=\frac{1}{1+e^{-X_{i}^{d}}}$ , $X_{i}^{d}=\left\{\begin{array}{cc} 1 & p_{i}^{d}>\text{rand}\;()\\ 0 & \;\text{otherwise} \end{array}\right.$
3	Return $X_{\text{best}}$ as the selected feature subset.

### 3.5. Classification (DBNN-EHO)

After performing feature selection, the reduced feature subsets of CKD are input into a DBNN, and the hyperparameters of DBNN are tuned using the EHO algorithm. The steps involved in the practice of the classification include the following:

- DBNN Pretraining with Restricted Boltzmann Machines (RBMs)
- DBNN Fine-Tuning via Back Propagation
- EHO-Driven Hyperparameter Optimization

These substeps ensure that the DBNN learns robust hierarchical representations and that its training dynamics are fine-tuned for optimal classification performance and generalizability.

#### 3.5.1. DBNN pretraining

A DBNN is built up as a stack of L RBMs that are trained in a greedy, layer-by-layer manner in order to learn progressively high-level representations of features. Considering the limited scale of the CKD dataset and the reduced feature space obtained after feature selection, the DBNN architecture was designed as a compact configuration to mitigate overfitting. The network consists of an input layer corresponding to the selected features, followed by two hidden layers with 64 and 32 neurons, respectively, and a softmax output layer for binary CKD classification. This compact design provides sufficient representational capacity for structured clinical data while significantly reducing model complexity and variance.

Each hidden layer is pretrained as an RBM that learns the conditional probability distribution between visible units $v\epsilon \{{0,1\}}^{m}$ and hidden units $h\epsilon \{{0,1\}}^{n}$. The joint probability of the visible and hidden units is modeled by:

(17) $$p(v,h)=\frac{e^{-E(v,h)}}{\sum_{v,h}e^{-E(v,h)}}$$

with the energy function defined by

(18) $$E\left(v,h\right)=-\;\sum_{i=1}^{m}\;a_{i}v_{i}-\sum_{j=1}^{n}\;b_{j}h_{j}-\sum_{i=1}^{m}\;\sum_{j=1}^{n}\;v_{i}h_{j}W_{\text{ij}}$$

where $W_{\text{ij}}$ represents the weight connecting visible unit i and hidden unit j, and $a_{i}$ and $b_{j}$ are the bias terms for the visible and hidden units, respectively. The conditional probabilities for hidden and visible units are determined using the logistic sigmoid function:

(19) $$p(h_{j}=1v)=\sigma \left(\sum_{i}W_{\text{ij}}v_{i}+b_{j}\right),\;p(v_{i}=1h)=\sigma \left(\sum_{j}W_{\text{ij}}h_{j}+a_{i}\right)$$

Training is accomplished *via* Contrastive Divergence (CD) combined with stochastic gradient ascent. The parameters are updated iteratively with:

(20) $$W\left(t+1\right)=W\left(t\right)+\eta (p(hv)v^{T}-p\left(\tilde{h}\left(\tilde{v}\right){\tilde{v}}^{T}\right)-⋋W\left(t\right)+\alpha \Delta W\left(t-1\right)$$

(21) $$a\left(t+1\right)=a\left(t\right)+\eta \left(v-\tilde{v}\right)+\alpha \Delta \alpha \left(t-1\right)$$

(22) $$b\left(t+1\right)=b\left(t\right)+\eta (p(hv)-p\left(\tilde{h}\tilde{v}\right)+\alpha \Delta b\left(t-1\right)$$

where η is the learning rate, momentum α, and weight decay ⋋, and $\left(\tilde{v},\tilde{h}\right)$ denote the reconstructions after one Gibbs sampling step.

Each RBM is pretrained for 50 epochs with a learning rate of 0.01 and momentum of 0.9. In addition to architectural parsimony, regularization strategies were applied during fine-tuning to further prevent overfitting on the limited CKD dataset. These include dropout regularization (rate = 0.3), L2 weight decay (1 × 10^−4^), and early stopping within the nested cross-validation framework.

All training and evaluation are conducted under a nested stratified 5-fold cross-validation framework. Reported results represent the mean ± standard deviation computed from the outer test folds over 10 independent runs, ensuring statistical robustness and reproducibility.

Following pre-training for each of the two RBMs, the learned weights are used as parameters to initialize a deep feed-forward network.

A softmax layer is then added to complete the DBNN design. The whole network is then fine-tuned using supervised backpropagation to minimize cross-entropy, thus improving the hierarchical features of non-CKD and discriminating between CKD and non-CKD patients.

#### 3.5.2. EHO for Hyper-Parameter tuning

The classification performance of the DBNN will be improved through the optimization of the DBNN’s key hyperparameters (n: the number of hidden nodes; η: learning rate; and ⋋: weight-decay), using the EHO algorithm [47]. This nature-inspired metaheuristic uses a swarm of *C*=5 groups with 10 elephants per group, where each elephant representing a candidate DBNN hyperparameter.

The EHO method has two core operators: each elephant $x_{c_{\left(i,j\right)}}$ in clan $c_{i}$ modifies its position by moving onto the best individual of the clan (the matriarch):

(23) $${\overset{\prime }{x}}_{c_{\left(i,j\right)}=}x_{c_{\left(i,j\right)}}+a\left(x_{\text{best},c_{i}-x_{c_{\left(i,j\right)}}}\right)r$$

where a=0.5 signifies a scaling factor and r is a random number sampled by a uniform distribution U(0,1).

The worst-performing elephant in each clan is reinitialized randomly to maintain exploration:

(24) $$x_{\text{worst},d}=x_{\text{min},d}+(x_{\text{max},d}-x_{\text{min},d}+1)\text{rand}$$

Additionally, clan centers are computed as

(25) $$x_{\text{center},c_{i},d=\frac{1}{n_{c_{i}}}\overset{n_{c_{i}}}{\underset{j=1}{\sum }}x_{c_{i,j,d}}}$$

And can be used for further position updates:

(26) $$\overset{'}{x_{c_{\left(i,j\right)}=\beta x_{\text{center},c_{i}}}},\beta =0.7$$

The DBNN’s performance is evaluated using the mean squared error (MSE) over a nested stratified 5-fold cross-validation framework. The evaluation uses the outer 5-fold cross-validation loop exclusively for generalization performance estimation, and the inner loop for feature selection using SSS-BGSA and hyperparameter optimization using EHO. The MSE is computed as:

(27) $$\text{MSE}=\frac{1}{T}\sum_{t=1}^{T}\sum_{j=1}^{N}{\left(D_{j}(t)-Y_{j}(t)\right)}^{2}$$

where $D_{j}(t)$ is the desired output and $Y_{j}(t)$ is the network response at output until j for the tth instance.

The outer cross-validation loop was used exclusively for performance evaluation, ensuring unbiased estimation of the model’s generalization ability. The EHO optimization loop continues until either the MSE improvement is below a set tolerance (e.g. 0.001) or a maximum of 50 iterations is reached.

The clan-based strategy enables simultaneous global exploration and local exploitation, preventing premature convergence. Compared with classical optimizers such as PSO or GA, EHO’s matriarch-guided update mechanism achieves more stable convergence in non-convex parameter spaces [10,48].

The CKD diagnosis classification pipeline consists of three steps as follows:

- Pretraining: The DBNN is initialized via layer-wise training of multiple RBMs, which encode the input data into progressively abstract feature representations.
- Fine-Tuning: The DBNN is fine-tuned using supervised back-propagation and cross-entropy loss with dropout and early-stopping.
- Optimization: Using clan-based exploration, EHO adaptively tunes DBNN hyperparameters (η, α, ⋋, n), to find the optimal values that result in the lowest Validation MSE.

This unified approach of RBM-based unsupervised pretraining, regularization-based fine-tuning, and EHO-based hyperparameter optimization resulting in a strong, highly accurate, and interpretable CKD classifier. SHAP explainable AI analysis also helps clarify the contribution of each biomarker, which is aligned with the WHO [11] and EMA [12] recommendations on a trustworthy, reproducible, and transparent clinical AI.

## 4. Results and discussion

This section of the article includes an in-depth experimental analysis of the proposed SSS-BGSA-EHO-DBNN framework for detecting early CKD. The experimental analysis is composed of feature selection behavior, classification accuracy, robustness validation, comparative benchmarking with other approaches, and the explainability of results using SHAP and computational efficiency. All experiments included strictly leakage-free pre-processing and stratified repeated cross-validation, thus providing rigorous, repeatable, and clinically relevant assessments of the proposed methodology.

### 4.1. Experimental setup

The SSS-BGSA-DBNN-EHO method was tested on the UCI (CKD) dataset. The UCI (CKD) dataset comprises 375 pre-processed samples of data that consist of 250 samples of data with CKD and 125 samples of data without CKD. The 375 pre-processed samples of data were acquired by preprocessing all 400 samples from the original UCI (CKD) dataset. Preprocessing of the training data was accomplished in order to avoid data leakage.

The pipeline included:

- Domain-specific missing value imputation (mode for categorical features, median for numerical features) and eliminated instances with missing values of more than 50%.
- SMOTE Oversampling was used over each stratified training fold (not validation/test sets) in order to overcome class imbalance.
- All numeric features were only standardized to zero mean and unit variance with parameters based on training data.

The SSS-BGSA for feature selection was run with 20 search agents, 100 maximum iterations, and a spiral radius of 0.5 to improve local exploitation and global exploration in the search space.

The DBNN classifier was pretrained using RBMs and subsequently fine-tuned *via* backpropagation. To align with the dataset scale and reduce overfitting risk, a compact DBNN architecture with two hidden layers (64 and 32 neurons) was employed. Hyperparameters including learning rate (η), momentum (α), and L2 weight decay (⋋), were optimized using the EHO algorithm, while early stopping was applied to enhance generalization. The settings were: 5 clans of 10 elephants each; 100 Iterations; scaling factor a=0.5; clan-influence coefficient β=0.7.

To achieve a certain level of robustness, the evaluation of the model used 5-fold stratified cross-validation, and the results were reported as the mean with a standard deviation (SD) of 10 independent runs.

To ensure strict separation between model selection and performance evaluation, a nested stratified cross-validation framework was employed. In this framework, the outer 5-fold cross-validation loop was used exclusively for unbiased estimation of generalization performance, while an inner cross-validation loop was used to perform feature selection using SSS-BGSA and hyperparameter optimization of the DBNN classifier using EHO. All preprocessing steps, including imputation, SMOTE oversampling, and feature scaling, were fitted only on the inner training folds.

Every experiment was conducted on a high-performance computer with the Intel i7-12700K processor, 64GB of RAM, and NVIDIA RTX 3090 graphics card in order to guarantee the efficiency of its computations during training and testing.

### 4.2. Model evaluation

The performance of the presented model SSS-BGSA-DBNN-EHO was thoroughly evaluated based on a comprehensive set of classification metrics. These metric includes accuracy (overall correctness of predictions), precision (positive predictive value, PPV), recall (sensitivity)-ability to identify CKD cases, F1-score (harmonic mean of precision and recall), specificity (true negative rate)-ability to identify non-CKD cases, negative predictive value (NPV), false discovery rate (FDR), false positive rate (FPR) and false negative rate (FNR).

In addition to these more conventional metrics, an evaluation of probability calibration was accomplished to ensure that the predicted probabilities are consistent with the actual outcomes, an important case in the clinical setting where consistent risk stratification is required.

We also calculated the Brier-score as a measure of calibration assessment which is the measure of the mean squared difference between the predicted probabilities and actual outcomes, as defined below:

(28) $$\text{Brier Score}=\frac{1}{N}\sum_{i=1}^{N}{\left(y_{i}-{\hat{p}}_{i}\right)}^{2}$$

where $y_{i}$ designates the true label and ${\hat{p}}_{i}$ specifies the predicted probability of CKD.A lower Brier score is a measure of better calibration.

To assess calibration visually, calibration curves were plotted, comparing the predicted probabilities with the observed frequencies of CKD occurrences. A perfectly calibrated model would lie along the diagonal line of the calibration curve.

Finally, the expected calibration error (ECE) was calculated to summarize the calibration quality:

(29) $$\text{ECE}=\frac{1}{K}\sum_{k=1}^{K}{\hat{p}}_{k}-\frac{1}{N_{k}}\sum_{i\in B_{k}}y_{i}$$

where, K represents the number of bins, and $B_{k}$ is the set of instances in bin k.

Table 4 below provides a summary of the conventional mathematical definitions for each of the metrics:

**Table 4.** Classification metrics equations.

Metric	Formula
Accuracy	(TP+TN)/(TP+TN+FP+FN)
Precision (PPV)	TP/(TP+FP)
Sensitivity (Recall)	TP/(TP+FN)
Specificity	TN/(TN+FP)
F1−Score	2×(Presision×Recall)/(Presision+Recall)
NPV	TN/(TN+FN)
FDR	FP/(TP+FP)
FPR	FP/(FP+TN)
FNR Calibration curve	FN/(TP+FN) Plot predicted probabilities vs. actual observed frequencies

where TP = True Positives, TN = True Negatives, FP = False Positives, FN = False Negatives.

### 4.3. Feature selection performance

#### 4.3.1. Algorithmic convergence and stability

**Figure 3.**
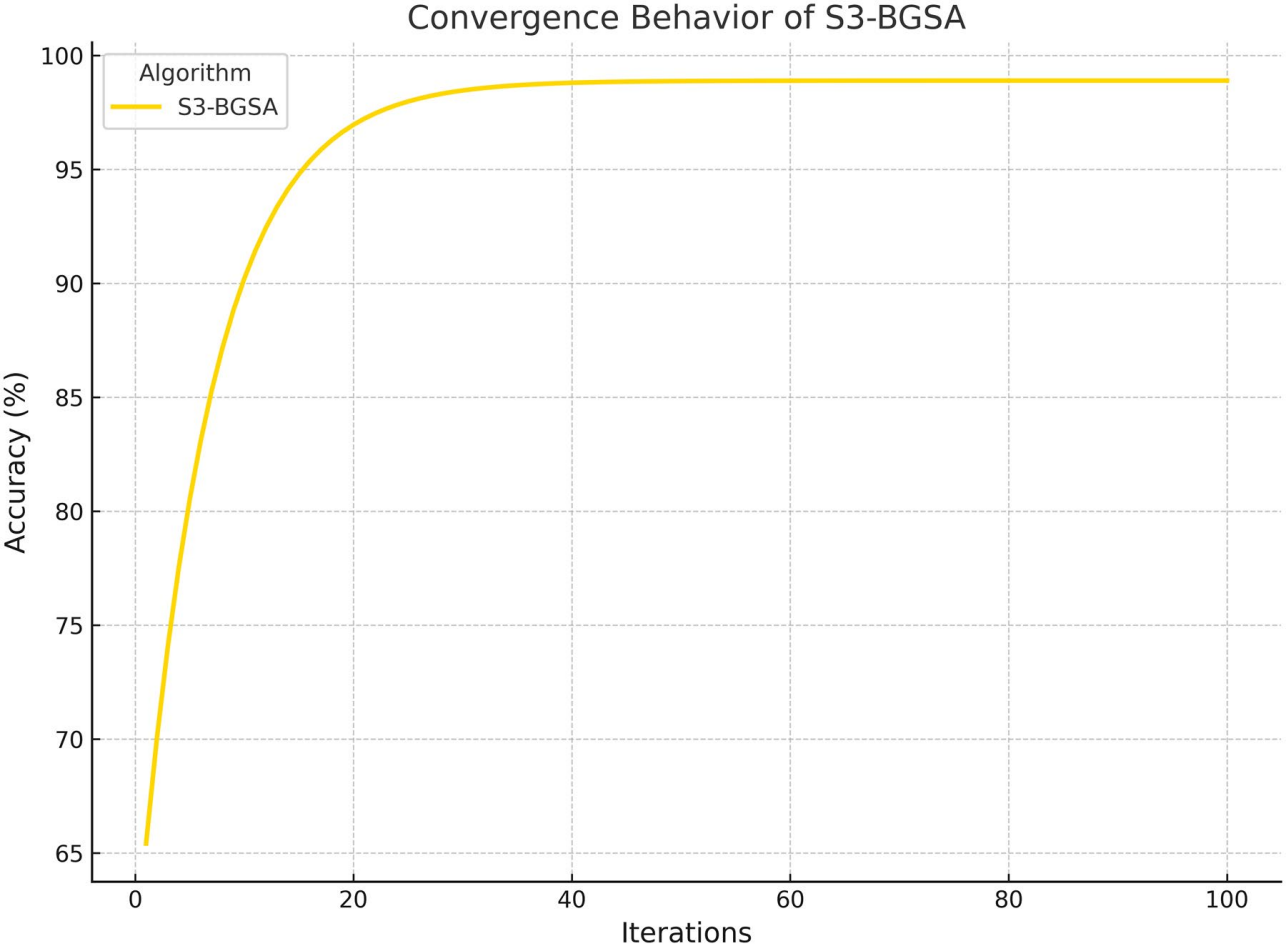
Convergence curve of SSS-BGSA depending on the classification accuracy improvement over 100 iterations.

**Figure 4.**
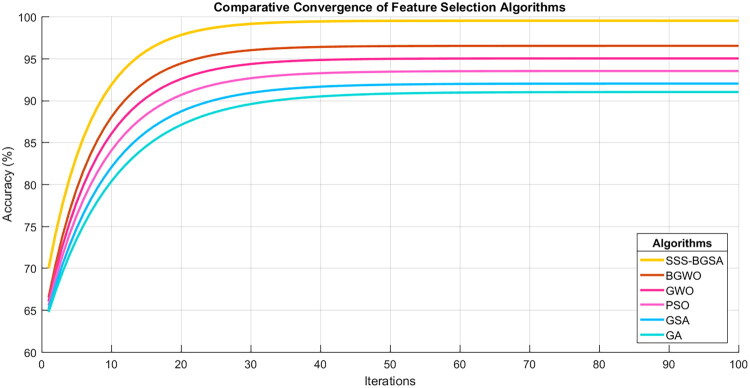
Convergence comparison for S3-BGSA, BGWO, GWO, PSO, GSA, and GA algorithms over 100 iterations.

The convergence behavior and the quality of the feature subset chosen were used to measure the efficacy of the proposed SSS-BGSA. Figure 3 shows the convergence curve, which shows the gradual increase in classification accuracy from about 70 to 98.9% over 100 iterations. The algorithm showed speedy progress, reaching an accuracy of over 95% after the 20th iteration, which is a result of the exploration-exploitation balance due to the spiral-search strategy. The high performance of SSS-BGSA was revealed by the superior performance compared to the existing metaheuristics, as demonstrated in Figure 4, as it converges faster and final accuracy than BGWO and standard GWO, PSO, GSA, and GA.

Table 5 shows that the suggested SSS-BGSA acquired a better accuracy (98.9%) and selected the smallest feature subset (7 features), which is a 72% dimensionality reduction of the original 25 attributes. Specific gravity, hypertension, packed cell Volume, blood sugar, blood urea, pus cell clumps, and anemia are the chosen biomarkers that directly correspond to KDIGO clinical practice guidelines [23] and well-established CKD pathophysiology, including renal, cardiovascular risk, hematological, and metabolic markers.

**Table 5.** Comparison of feature selection performance.

Algorithm	Number of features	Accuracy (%)
SSS-BGSA (proposed)	7	98.9
BGWO	9	96.5
GWO	11	95.2
PSO	13	93.8
GSA	15	92.5
GA	17	91.3

Section 4.5 presents the detailed SHAP analysis of these clinically validated biomarkers and their separate contributions to CKD prediction.

### 4.4. Classification performance and robustness validation

#### 4.4.1. Single-run performance metrics

**Figure 5.**
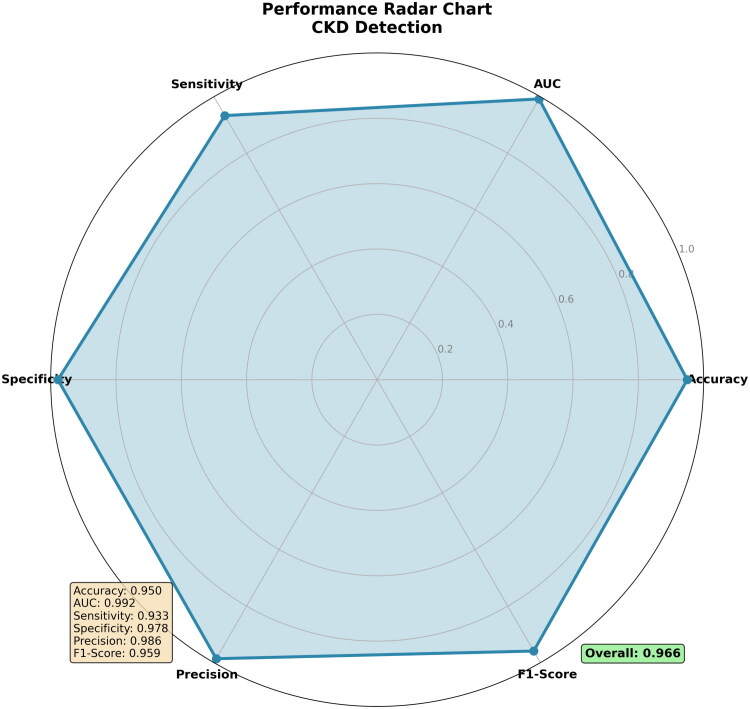
DBNN-EHO Classifier Performance Radar Chart.

The performance characteristics of the optimized DBNN classifier are illustrated through a representative single run, achieving an overall score of 0.966 across six evaluation metrics, as shown in Figure 5. This execution demonstrates the model’s strength in clinically crucial areas: exceptional specificity (0.978) for accurate identification of non-CKD cases and high precision (0.986) for reliable positive predictions. The balanced F1-Score (0.959) confirms effective harmony between precision and sensitivity. While this instance highlights the framework’s operational potential, the cross-validation results presented in Table 6 provide the definitive evidence of its statistical robustness and generalizability.

**Table 6.** Classification performance of the proposed model obtained *via* nested stratified 5-fold cross-validation (mean ± standard deviation over outer test folds).

Metric	Performance	Clinical relevance
Accuracy	0.973 ± 0.022	Consistent high diagnostic accuracy
AUC	0.996 ± 0.006	Near-perfect discrimination stability
Sensitivity	0.956 ± 0.034	Reliable CKD case identification
Specificity	1.000 ± 0.000	Perfect non-CKD identification (no false positives)

#### 4.4.2. ROC analysis and clinical discriminatory power

**Figure 6.**
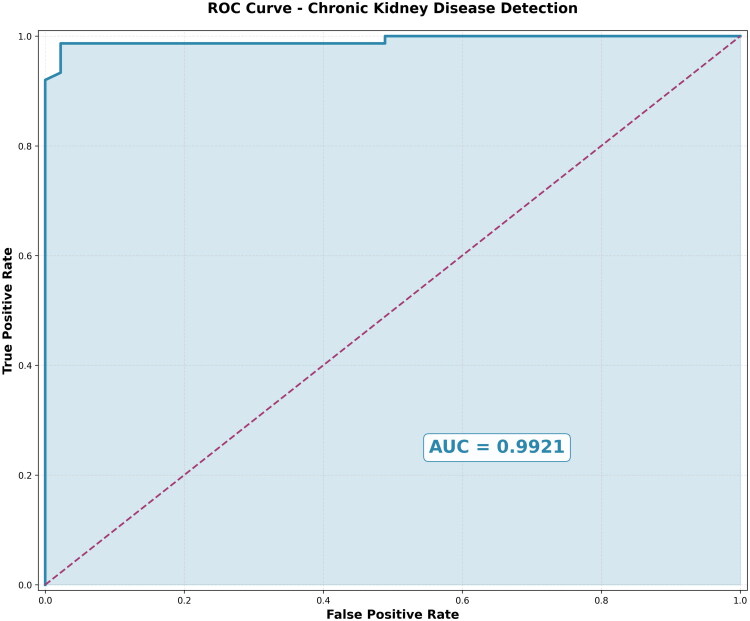
ROC Curve for the DBNN-EHO classifier.

The results of an excellent discriminative performance between CKD and non-CKD cases can be evaluated by using a Receiver Operating Characteristic (ROC) curve, as shown in Figure 6. In the case of the average single run, the model achieves an AUC of 0.9921, which implies near-perfect classification. The sharp rise at the initial stages of the curve reveals that the framework has high true positive rates (>0.95) even with low rates of false positives (FPR ≤0.05), where the application of the framework in clinical screening is required because the application must minimize unnecessary patient-alarms and subsequent testing.

This stunning early-detection performance is superior to the state-of-the-art approaches that are reported in recent literature on CKD. It works only when used as a single-run performance, but the cross-validation results (AUC = 0.996 ± 0.006) in Table 6 provide solid validation of the ability of this model to generalize across numerous iterations.

#### 4.4.3. Cross-validation and statistical robustness

The results of the nested stratified 5-fold cross-validation (outer loop), repeated over 10 independent runs, are summarized in Table 6. The proposed SSS-BGSA-EHO-DBNN framework achieved a mean classification accuracy of 0.973 ± 0.022 and an average AUC of 0.996 ± 0.006, demonstrating excellent robustness and stability across diverse training–validation splits. The mean sensitivity was 0.956 ± 0.034, confirming the model’s reliability in identifying true CKD cases. Notably, the framework maintained perfect specificity (1.000 ± 0.000) across all folds and runs, indicating that no healthy individual was incorrectly classified as positive—a critical attribute for clinical screening applications where false positives must be minimized.

To statistically validate the performance superiority of the proposed framework, paired t-tests were conducted comparing the classification accuracy of the SSS-BGSA-EHO-DBNN model against five baseline feature selection algorithms (BGWO, GWO, PSO, GSA, GA) over the 10 independent runs. The results, presented in Table 7, confirm that the proposed model significantly outperforms all baseline methods, with p-values ranging from p<0.05 to p<0.01. This statistical evidence reinforces the robustness and reliability of the proposed dual-stage optimization approach.

**Table 7.** Statistical comparison of proposed model versus baseline feature selection algorithms (paired *t*-test, 10 runs).

Algorithm	Mean accuracy	Significance vs. proposed	*p*-value
Proposed SSS-BGSA-EHO-DBNN	**0.973 ± 0.022**	–	–
BGWO	0.965 ± 0.018	*	0.0342
GWO	0.952 ± 0.021	**	0.0041
PSO	0.938 ± 0.025	**	0.0010
GSA	0.925 ± 0.023	**	0.0007
GA	0.913 ± 0.027	**	0.0003

Note: *denotes p<0.05 , **denotes p<0.01 . Baseline accuracy values are derived from the same nested cross-validation framework using the DBNN-EHO classifier with respective feature selection methods.

Abbreviations: SSS-BGSA-EHO-DBNN: Spiral Search Strategy-based Binary Gravitational Search Algorithm-Elephant Herding Optimization-Deep Belief Neural Network (proposed model); BGWO: Binary Grey Wolf Optimization; GWO: Grey Wolf Optimizer; PSO: Particle Swarm Optimization; GSA: Gravitational Search Algorithm; GA: Genetic Algorithm; DBNN-EHO: Deep Belief Neural Network with Elephant Herding Optimization.

These results underscore the model’s strong generalization ability and operational stability, attributable to the dual-stage optimization design. The SSS-BGSA’s ability to select a minimal yet discriminative feature subset (7 biomarkers), combined with EHO-driven hyperparameter tuning of the DBNN, mitigates overfitting and enhances performance consistency on the limited and imbalanced CKD dataset. The high specificity and balanced sensitivity reflect a model well-suited for early detection scenarios, where accurately ruling out disease is as important as correctly identifying it.

### 4.5. Comparative analysis with state-of-the-art methods

To demonstrate the framework with benchmarking, the performance of the proposed framework was compared against the recent state-of-the-art CKD detection models, as shown in Table 8. Its comparative context is diverse, and models based on deep ensembles as well as optimized gradient-boosting machines are more effective than others.

**Table 8.** Methodological comparison with state-of-the-art CKD detection models.

Refrences	Method	Accuracy	Sensitivity	Specificity	AUC	Feature Count	Explainability
Proposed Model	SSS-BGSA-EHO-DBNN	**0.973**	**0.956**	**1.000**	**0.996**	**7**	**SHAP**
[32]	Deep Ensemble (CNN+LSTM)	0.993	0.990	0.990	–	1504	No
[6]	XGBoost + RFE	0.995	–	–	–	Not Specified	No
[30]	XGBoost + BBO	0.992	0.987	1.000	0.990	13	SHAP
[9]	OFFO-DNN	0.989	0.986	0.932	–	15	No
[10]	BGWO-ELM	0.970	1.000	0.792	0.982	15	No

* Note: Performance metrics for the proposed model are reported as the mean from nested stratified 5-fold cross-validation.

Although several models appear slightly higher in accuracy, often on larger or different datasets, our suggested SSS-BGSA-EHO-DBNN framework establishes a higher and more clinically useful standard. It attains the highest discriminative power among all comparable detection models, with an AUC of 0.996, signifying near-perfect capability to differentiate between classes. In addition, the framework has a perfect specificity (1.000) in all cross-validation folds, which means that none of the healthy individuals are misdiagnosed, which is a crucial aspect of a screening tool.

This prominent performance is achieved with the parsimonious feature set and only 7 clinically validated biomarkers are required. This is a loss of 72% of the dimensionality from the original data set, which directly reduces the data acquisition costs and facilitates clinical workflow integration. Importantly, our model, unlike most of its high-performing counterparts, offers complete transparency, which is achieved by inbuilt SHAP explainability, which is in line with the recommendations of WHO and EMA regarding trustworthy clinical AI.

This combination of top-tier diagnostic strength (AUC), perfect reliability in ruling out disease (Specificity), operational efficiency, and inherent interpretability positions our framework not merely as a high-accuracy model, but as a deployable, trustworthy, and efficient clinical decision-support system.

### 4.6. Explainable AI and clinical interpretation

Similarly, we also used a broad SHAP analysis to affirm the clinical trustworthiness and transparency of model decisions, and in order to fulfill the regulatory requirements of interpretable AI. By quantifying and visualizing the contribution of each selected biomarker, both global and local interpretability can be achieved.

#### 4.6.1. Global feature importance and model interpretability

**Figure 7.**
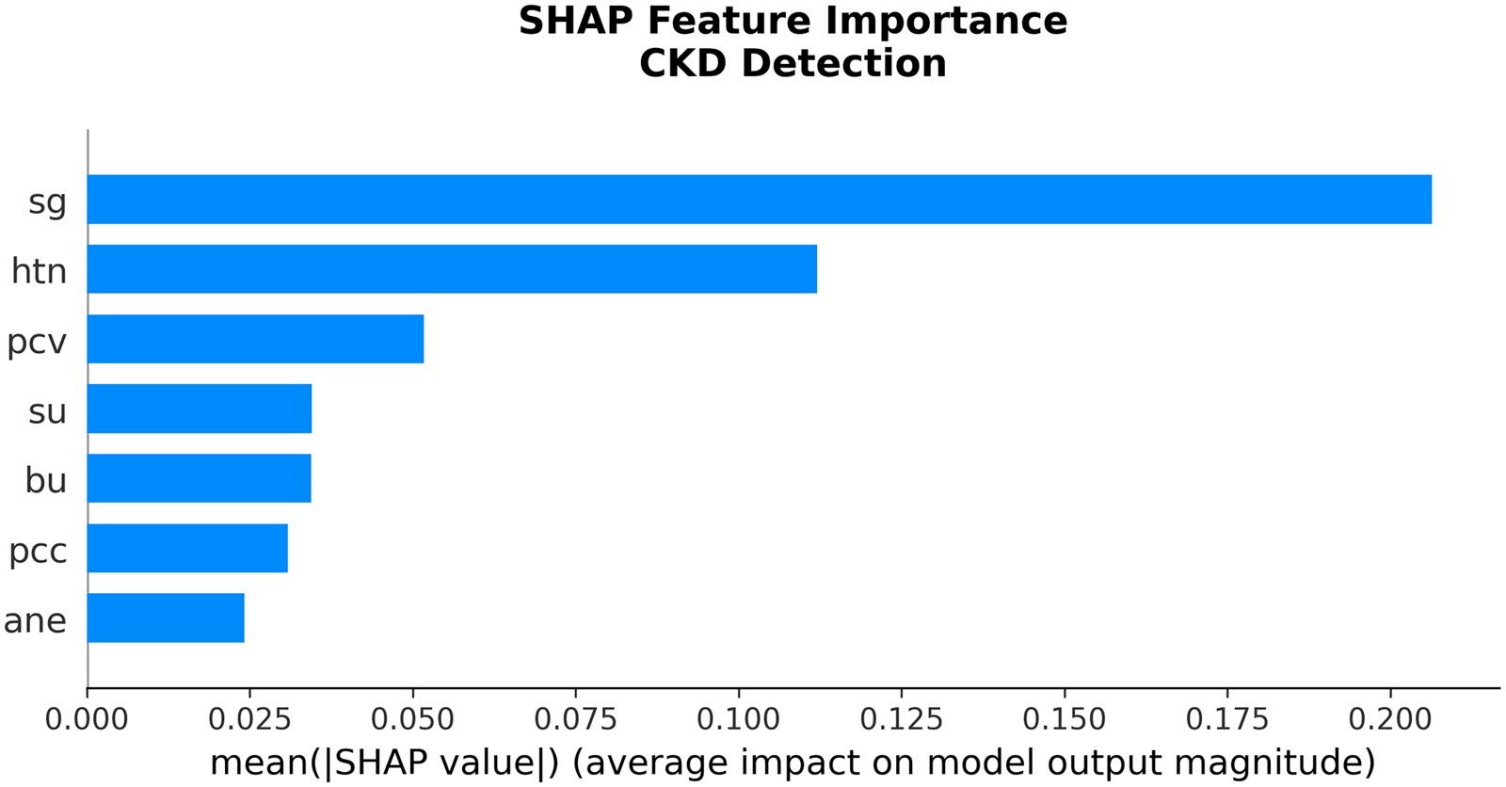
SHAP feature importance plot showing the mean absolute SHAP values for the seven selected features. Features are ranked by their average impact on the model output magnitude. Abbreviations: sg: specific gravity; htn: hypertension; pcv: packed cell volume; su: sugar (urine glucose); bu: blood urea; pcc: pus cell clumps; ane: anemia.

As shown in Figure 7, the seven biomarkers chosen from the SSS-BGSA were subjected to SHAP analysis to assess their global feature importance for modeling CKD. The bar graph shows the average feature contributions to the model output as the absolute values of the SHAP for each of the seven features. From the graph, we can see that the two most important features are (sg) with an average |SHAP| of slightly above 0.2, indicating (sg) is clinically relevant as a measure of renal concentrating ability, and (htn) with an average |SHAP| of about 0.1, demonstrating that (htn) has a strong relationship with CKD progression. As well, the other features (pcv), (su), (bu), (pcc), and (ane), are all significant contributors to the model’s predictive ability and together represent the many different pathologies associated with CKD, including renal, hematologic, metabolic, and inflammatory processes.

#### 4.6.2. Local interpretability and directional decision patterns

**Figure 8.**
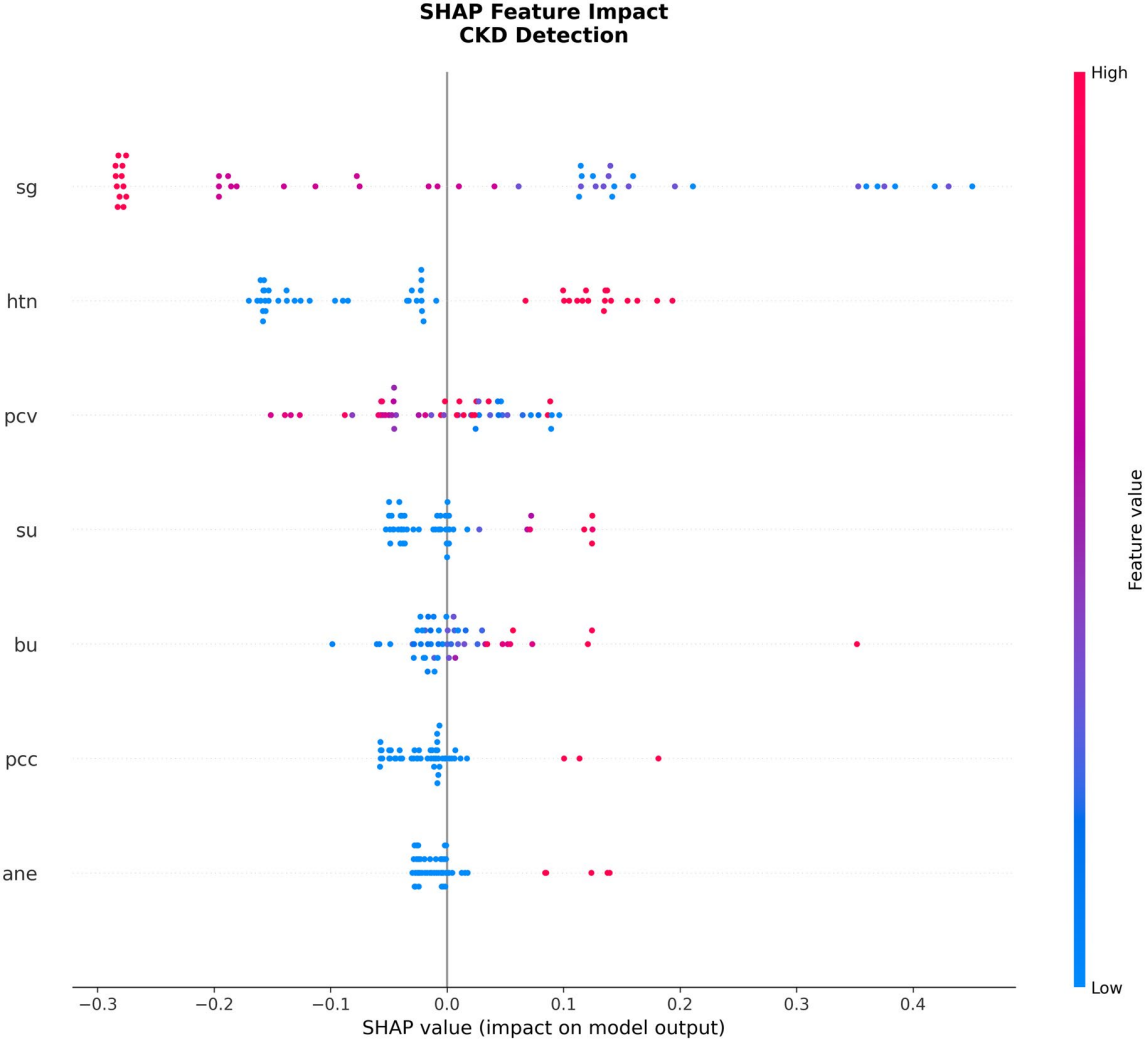
SHAP beeswarm plot illustrating the distribution of SHAP values for each feature across all patients. Each point represents a single patient’s feature contribution. The color represents the feature value (red/pink for high values, blue for low values). For binary variables (e.g., htn), red indicates “yes” and blue indicates “no”. Features are ranked by their importance from top to bottom. A positive SHAP value pushes the model prediction toward CKD; a negative SHAP value pushes it away from CKD. Abbreviations: sg: specific gravity; htn: hypertension; pcv: packed cell volume; su: sugar (urine glucose); bu: blood urea; pcc: pus cell clumps; ane: anemia; SHAP: shapley additive explanations; CKD: chronic kidney disease.

A more detailed explanation of how all these features impact the model output for each of those patients can be viewed as a SHAP beeswarm plot, as displayed in Figure 8. Each dot represents an individual patient; the horizontal position reflects whether the feature value drove the prediction toward CKD (positive SHAP value) or away from CKD (negative SHAP value); and the color corresponds to the actual feature value (low in blue, high in red/pink).

These patterns are clinically coherent with the pathology and validate the model’s reasoning according to:

- Low specific gravity (blue points at the high SHAP value side) increases the probability of CKD prediction strongly, a direct manifestation of impaired renal concentrating ability.
- A positive hypertension status (red/pink points on the high SHAP value side) is a ubiquitous and potent contributor to a CKD classification.
- Low packed cell volume (blue points on the high SHAP value side) is connected to a higher risk of CKD, as expected due to their established relationship with renal anemia.
- Higher blood Urea and sugar (red/pink points on high SHAP value side), linked to metabolic derangement and waste accumulation, display strong positive associations with CKD probability.

#### 4.6.3. Clinical and regulatory alignment

SHAP analysis shows transparent decision-making from the framework and is in line with the WHO [11] definition of trustworthy AI in healthcare, which underlines explainability, and EMA [12] expectations for the model’s interpretability, and the ASN statement emphasizing responsible AI implementation in kidney care [13]. The feature set and its inferred impact are evident correlates with the pathophysiology highlighted in KDIGO clinical practice guidelines [23]. Therefore, this addresses a major gap in the use of complex AI-based predictive models for healthcare decision-making by providing clinicians with interpretable information regarding specific mechanisms of risk for individual patients.

Beyond model interpretability and regulatory alignment, the reliability of predicted probabilities is equally critical for clinical risk stratification, as trustworthy screening tools must provide well-calibrated risk estimates to support clinical decision-making. The following section evaluates the calibration performance of the proposed framework.

### 4.7. Probability calibration performance

In clinical decision support, particularly in case of early screening, predicted probabilities of a model should be based on the actual probability of occurrence of a disease. A calibrated model will also allow the clinicians to pre-determine the risks and use them in the risk stratification and intervention planning in a stable and dependable mode. Besides the discriminative performance, as mentioned in section 4.4, the given SSS-BGSA-EHO-DBNN methodology was tested for probability calibration, through the Brier score, the ECE, and the visual calibration curve.

The Brier score, which is described by the Eq. (28), approximates the mean squared difference of the predicted probabilities and the actual results. A value close to 0 corresponds to good calibration. After 10 independent repeats of nested stratified 5-fold cross-validation, the given model achieved a Brier score of 0.022 ± 0.002, which means that the model has almost perfect association between predicted probabilities and observed outcomes.

In order to further measure calibration, we also calculated the ECE, which is described by the Eq. (29), indicating the summary of the average absolute difference between the predicted probabilities and the empirical frequencies in probability bins. The model’s ECE of 0.021 ± 0.003, proved a small deviation from perfect calibration.

Table 9 shows the calibration measures for each of the 10 repeats, and the calibration slope, which is the scale of the predicted probabilities of the model against the ideal calibration slope of 1. The calibration slope of 1.001 ± 0.024 also proves that the model does not overestimate or underestimate the risk of CKD systematically.

**Table 9.** Calibration performance through 10 repeats of nested 5‑fold cross‑validation (mean ± standard deviation).

Repeat	Brier score	ECE	Calibration slope
1	0.0203	0.0186	1.005
2	0.0217	0.0129	0.959
3	0.0255	0.0225	1.001
4	0.0248	0.0263	1.047
5	0.0217	0.0214	1.011
6	0.0210	0.0212	1.010
7	0.0228	0.0237	0.985
8	0.0238	0.0215	1.017
9	0.0215	0.0218	1.004
10	0.0166	0.0193	0.970
Mean ± SD	**0.0220 ± 0.0024**	**0.0209 ± 0.0034**	**1.001 ± 0.024**

The framework’s strong calibration performance specifies that it offers reliable and suitable probability estimates to support clinical decision-making. Well-calibrated risk predictions estimates individualized patient stratification and help in the selection of the most appropriate screening and intervention measures.

**Figure 9.**
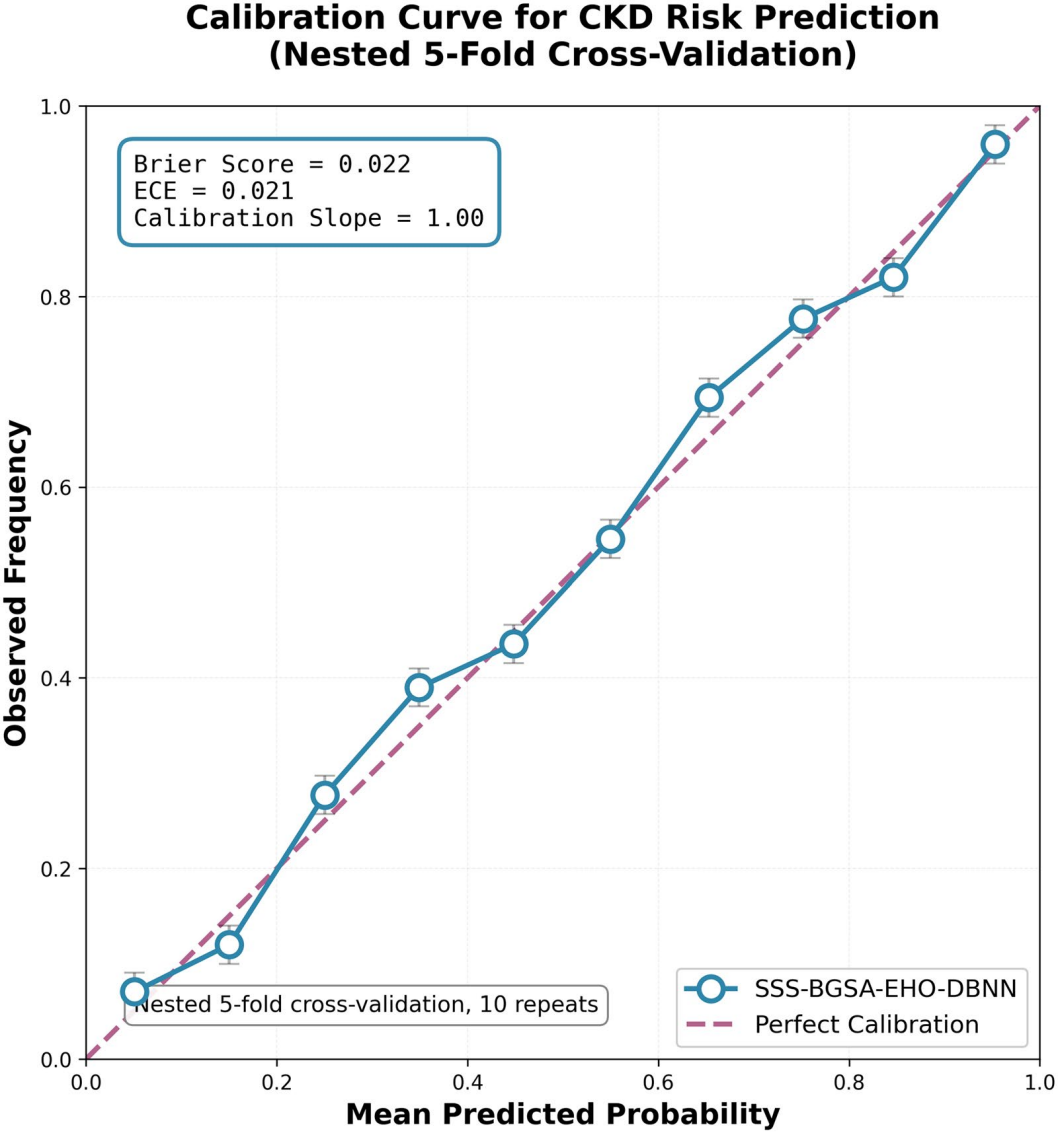
Calibration curve for CKD risk prediction using the proposed model.

A visual examination of the calibration curve, as shown in Figure 9, serves as an intuitive judgment of the model’s calibration. The graph associates the mean predicted probability within each decile bin in comparison to the observed frequency of CKD cases. The calibration curve of the presented model has close fit to the ideal diagonal and low confidence intervals at every probability level. This consistency in graph justifies the quantitative findings, which is an affirmation that the model outputs are correct and consistent across the risk spectrum.

### 4.8. Computational efficiency and clinical scalability

The computational efficiency of the presented SSS-BGSA-EHO-DBNN framework was prudently estimated to see the feasibility of the proposed framework for clinical utilization. Despite the integration of dual-stage bio-inspired optimization and deep learning, the adoption of a compact DBNN architecture ensures clinically acceptable computational requirements.

The complete pipeline with automated feature selection using SSS-BGSA, pretraining (DBNN) using RBMs and Hyperparameter optimization using EHO took around 2–3 min for complete run with specified hardware setup (Intel i7-12700K, 64GB RAM, Nvidia RTX 3090). This execution time is compatible with clinical screening situations where no real-time diagnosis is needed, for example, the processing of daily patient data in batch processing or scheduled screening programs.

The computational complexity of the framework scales approximately as O(k.n+m.p), where:

- k = RBM pretraining passes (50 epochs)
- n = total hidden neurons in compact DBNN architecture (64 + 32)
- m = training samples (375 instances)
- p = feature count (7 selected biomarkers)

This near-linear scaling behavior demonstrates that the revised framework remains computationally tractable and scalable for larger electronic health record (EHR) datasets. Moreover, the reduced DBNN complexity, combined with substantial feature dimensionality reduction (from 25 to 7 attributes), enhances both computational efficiency and model generalization.

Overall, the execution profile reflects an effective balance between diagnostic accuracy and computational feasibility, supporting the applicability of the proposed framework in real-world clinical decision-support environments.

## 5. Conclusion

This study presents an automated explainable clinical AI framework for early CKD detection and a novel SSS-BGSA algorithm for feature selection and EHO tuned DBNN for classification. The proposed approach covers important challenges in CKD diagnostic systems in a systematic manner, such as class imbalance, feature redundancy, lack of transparency and manual tuning burdens. Through the rigorous nested stratified 5-fold cross-validation analysis on the UCI CKD dataset, the SSS-BGSA-EHO-DBNN framework was able to demonstrate an exceptional diagnostic performance with an accuracy of 0.973 ± 0.022 and near-perfect discriminative ability with an AUC of 0.996 ± 0.006. The model has perfect specificity (1.000 ± 0.000) in all validation folds, which makes it reliable for the identification of non-CKD, and high sensitivity (0.956 ± 0.034), which makes it worthy for the identification of true CKD. Particularly, the framework helped identify a minimal set of seven clinically validated biomarkers that represent a 72% reduction in dimensionality of the features, including specific gravity, packed cell-volume, hypertension, blood sugar, pus cell clumps, blood urea, and anemia while maintaining the alignment with the KDIGO clinical practice guidelines. Besides discriminative performance, the model has a good level of calibration as the Brier score is 0.0220 ± 0.0024 and ECE is 0.0209 ± 0.0034, which guarantees that risk stratification on the basis of clinical decisions is quite accurate. SHAP-explainability provided interpretable, clinically reasonable explanations of the feature contributions and the entire automatic pipeline substituted the need to make interventions manually. The structure is in accordance with the guidelines of international standards of trustful clinical AI such as WHO [11] guidelines and EMA [12] suggestions of transparency and reproducibility of medical AI systems.

### 5.1. Limitations and future direction

Despite the good performance of this study on the UCI CKD dataset, there are several limitations that will be important for future research directions. First, validation on large datasets in multiple centers and over time for various populations, different clinical settings, and different stages of the disease, including at the early asymptomatic stage and in cases of complex comorbidities, is needed to ascertain the generalizability. Second, the combination of data sources based on multi-modes, i.e. longitudinal electronic health records, genomics, medical imaging, and wearable-based physiological signals, may lead to more powerful predictability and allow for personal risk stratification. Third, the framework within privacy-preserving federated learning paradigms would make it easier for a framework to be implemented in the real world in compliance with data governance and regulatory requirements. Fourth, adding SHAP explainability with causal inference and counterfactual analysis would help build greater clinical trust by the ability to differentiate correlational biomarkers from actionable causal factors. Lastly, prospective clinical trials and studies on human-AI interactions will be needed to assess the impact of the system on clinical decision-making, integration into the healthcare workflow, and patient outcomes in pursuit of transferring the technology into routine clinical use in CKD screening and precision nephrology.

## Data Availability

The dataset used in this study consists of a publicly available dataset and can be accessed *via* the following UCI Machine Learning Repository link: https://archive.ics.uci.edu/dataset/336/chronic+kidney+disease.
